# Topical insulin-like growth factor 1 treatment using gelatin hydrogels for glucocorticoid-resistant sudden sensorineural hearing loss: a prospective clinical trial

**DOI:** 10.1186/1741-7015-8-76

**Published:** 2010-11-25

**Authors:** Takayuki Nakagawa, Tatsunori Sakamoto, Harukazu Hiraumi, Yayoi S Kikkawa, Norio Yamamoto, Kiyomi Hamaguchi, Kazuya Ono, Masaya Yamamoto, Yasuhiko Tabata, Satoshi Teramukai, Shiro Tanaka, Harue Tada, Rie Onodera, Atsushi Yonezawa, Ken-ichi Inui, Juichi Ito

**Affiliations:** 1 Department of Otolaryngology, Head and Neck Surgery, Graduate School of Medicine, Kyoto University, Kyoto, Japan; 2 Department of Otolaryngology, Faculty of Medicine, University of Tokyo, Tokyo, Japan; 3 RIKEN Center for Developmental Biology, Kobe, Japan; 4 Department of Biomaterials, Institute for Frontier Medical Sciences, Kyoto University, Kyoto, Japan; 5 Department of Clinical Trial Design & Management, Translational Research Center, Kyoto University Hospital, Kyoto, Japan; 6 Collaboration Center for Community and Industry, Sapporo Medical University, Sapporo, Japan; 7 Department of Pharmacy, Kyoto University Hospital, Kyoto, Japan

## Abstract

**Background:**

Sudden sensorineural hearing loss (SSHL) is a common condition in which patients lose the hearing in one ear within 3 days. Systemic glucocorticoid treatments have been used as standard therapy for SSHL; however, about 20% of patients do not respond. We tested the safety and efficacy of topical insulin-like growth factor 1 (IGF1) application using gelatin hydrogels as a treatment for SSHL.

**Methods:**

Patients with SSHL that showed no recovery to systemic glucocorticoid administration were recruited. We applied gelatin hydrogels, impregnated with recombinant human IGF1, into the middle ear. The primary outcome measure was the proportion of patients showing hearing improvement 12 weeks after the test treatment. The secondary outcome measures were the proportion of patients showing improvement at 24 weeks and the incidence of adverse events. The null hypothesis was that 33% of patients would show hearing improvement, as was reported for a historical control after hyperbaric oxygen therapy.

**Results:**

In total, 25 patients received the test treatment at a median of 23 days (range 15-32) after the onset of SSHL, between 2007 and 2009. At 12 weeks after the test treatment, 48% (95% CI 28% to 69%; *P *= 0.086) of patients showed hearing improvement, and the proportion increased to 56% (95% CI 35% to 76%; *P *= 0.015) at 24 weeks. No serious adverse events were observed.

**Conclusions:**

Topical IGF1 application using gelatin hydrogels is well tolerated and may be efficacious for hearing recovery in patients with SSHL that is resistant to systemic glucocorticoids.

## Background

Sudden sensorineural hearing loss (SSHL) is a condition in which an individual experiences hearing loss of at least 30 dB over at least three test frequencies in one ear within a period of 3 days [1]. Some patients recover completely without medical intervention, often within the first 3 days. Others get better slowly over a 1-week or 2-week period, which is known as 'spontaneous recovery' [1]. Although a good recovery is likely, 15% of patients with SSHL experience hearing loss that worsens over time. Approximately 40,000 new cases of SSHL occur each year in the US [1], and 35,000 patients with SSHL consult a doctor each year in Japan [2]. SSHL can affect anyone; however, for reasons that so far remain unknown, it is most often reported in people aged between 30 and 60 years. The most common therapy for SSHL is the systemic application of glucocorticoids. Unfortunately, about 20% of patients do not respond to this treatment [3].

Based on these findings, researchers have sought alternative therapeutic options for SSHL. Protecting auditory hair cells and primary neurons from irreversible degeneration is a practical strategy, as inner ear cells have limited regeneration capacity [4]. Recent improvements in our understanding of the role of growth factors in the maintenance of mature peripheral auditory systems have led to numerous attempts to define ways to reduce auditory hair cell and neuron degeneration, which have indicated that some growth factors have potential for the treatment of SSHL [5-8]. However, growth factors have not yet been used for this purpose in a clinical setting, as several obstacles have hindered their progress. Safe and effective methods for the sustained delivery of growth factors to the inner ear need to be developed to facilitate their clinical application. As a solution to this problem, we used gelatin hydrogels as a vehicle to deliver growth factors to the inner ear [9]. Gelatin hydrogels consist of gelatin polymers that are electrostatically complexed with growth factors [10]. The growth factors are released by the enzymatic degradation of the gelatin polymers after application. Our focus was on insulin-like growth factor 1 (IGF1), which has been approved for clinical application. We conducted a series of animal experiments, which revealed that topical IGF1 application via gelatin hydrogels significantly improved hearing by protecting auditory hair cells against damage caused by intense noise exposure [11] or ischaemic injury [12]. Moreover, no adverse events were observed in animals following the local application of IGF1 via gelatin hydrogels [11].

Here, we report on a prospective clinical trial of topical IGF1 application through gelatin hydrogels for the treatment of glucocorticoid-resistant SSHL, which was intended to provide preliminary estimates of variables for generating hypotheses for more specific studies using randomised trials when appropriate. Systemic glucocorticoid application has been regarded as a primary treatment of choice for SSHL. We recruited patients with SSHL that showed no recovery to systemic glucocorticoid administration as subjects in the present study.

## Methods

### Patients

Patients were eligible for inclusion in the study if they met the following conditions: they had been diagnosed between December 2007 and July 2009 at the Department of Otolaryngology, Head and Neck Surgery of Kyoto University Hospital, Japan as having definite or probable SSHL within 29 days of onset; they presented with an abnormality in evoked otoacoustic emission, which indicated dysfunction of the auditory hair cells; no recovery was determined according to the criteria for hearing improvement as set by the Sudden Deafness Research Committee of the Japanese Ministry of Health, Labour and Welfare in 1984 [13] (Table 1) more than 7 days after systemic glucocorticoid treatment; and they were aged over 20 years. We excluded patients with active chronic otitis media, acute otitis media, otitis media with effusion or dysfunction of the auditory tube, a history of previous treatments (except for systemic application of glucocorticoids or prostaglandin E1), malignant tumours, severe liver dysfunction (aspartate aminotransferase (AST) >100 IU/L and alanine aminotransferase (ALT) >100 IU/L), uncontrolled diabetes (haemoglobin A1C (HbA1c) >10%), pituitary or adrenal dysfunction, severe systemic illness that affected life expectancy, a history of severe drug allergy, or a history of alcohol or drug dependence within the past 1 year, and pregnant or lactating women. Magnetic resonance imaging (MRI) was performed on all patients to rule out acoustic neurinoma.

**Table 1:** Criteria for hearing improvement determined by the Sudden Deafness Research Committee of the Japanese Ministry of Health, Labour and Welfare in 1984

Improvement	Criteria
Complete recovery	Recovery of a hearing level within 20 dB at all five frequencies tested (0.25, 0.5, 1.0, 2.0 and 4.0 kHz) or recovery to the same level as the opposite side in pure tone auditometry

Marked recovery	More than 30 dB recovery in the mean hearing level at the five frequencies tested

Slight recovery	Recovery of 10 to 29 dB in the mean hearing level at the five frequencies tested

No recovery	Less recovery than 10 dB in the mean hearing level at the five frequencies tested

This study was single arm, non-randomised and open. Placebo applications and blinding were not used, as it was anticipated that they would have reduced compliance.

The primary outcome measure was the proportion of patients showing hearing improvement, which was defined as better than slight recovery according to the criteria shown in Table 1, 12 weeks after the test treatment. The secondary outcome measures were the proportion of patients showing hearing improvement 24 weeks after the test treatment and the incidence of adverse events during the observation period.

### Ethics approval

This study was conducted in accordance with the Declaration of Helsinki and its amendments, and approved by the Ethical Committee of the Graduate School of Medicine, Kyoto University (registered number, C165). Each patient gave written, informed consent to participate in this study.

### Trial registration

This trial was registered in the University Hospital Medical Information Network Clinical Trials Registry (UMIN-CTR) on 6 December 2007 under trial registration number UMIN-CTRR000000936.

### Procedures

The test treatment was performed within 4 days of registration. Gelatin hydrogels were made from porcine skin gelatin (Nitta Gelatin Inc., Osaka, Japan) in a clean room at the Department of Pharmacy, Kyoto University Hospital, according to the method described previously [14], and were preserved at temperatures below 4°C before use. Procedures for topical IGF1 treatment were performed in the Day-Surgery Unit of Kyoto University Hospital. Mecasermin (recombinant human IGF1 (Somazon), 10 mg injection; Astellas Pharma Inc., Tokyo, Japan) was dissolved in physiological saline at a final concentration of 10 mg/ml. A 30 μl sample of mecasermin solution was mixed with 3 mg of gelatin hydrogels 60 min before application. The hydrogel containing 300 μg of mecasermin was placed in the round-window niche of the middle ear following tympanostomy under local anaesthesia with 1% lidocaine. A single application was used. Patients were hospitalised for 4 days after the surgical procedure, and their general and local conditions were examined at the outpatient clinic of the Department of Otolaryngology, Head and Neck Surgery, Kyoto University Hospital, for 24 weeks after the test treatment. Pure-tone audiometry and evoked otoacoustic emission were measured on the day of registration, at 3 days after the test treatment, and at 1, 2, 4, 12 and 24 weeks after the test treatment. During the observation period, all adverse events were recorded.

### Statistical analysis

The threshold improvement (33%, 66/199) was based on a historical control of hyperbaric oxygen therapy (19 times in total; range 5-55) for 199 patients with glucocorticoid-resistant SSHL at Kyoto University Hospital between October 2000 and September 2006 [15]. The null hypothesis was that the proportion of patients with hearing improvement at 12 or 24 weeks after the test treatment would be equivalent to the proportion of patients with hearing improvement reported in a historical control administered hyperbaric oxygen therapy. The sample size was based on binominal distribution with a one-sided significance level of 0.05 and a power of 0.90 (expected proportion of 63%). The required sample size was 25 after considering 10% (3 samples) of patients who would be excluded from the analysis. The null hypothesis was rejected at the 0.05 level of probability (one-sided) based on a binominal distribution. Statistical analyses were performed using SAS v.9.2 (SAS Institute Inc. Cary, NC, USA).

## Results

In all, 26 patients fulfilled the inclusion criteria, 1 of whom was excluded before the test treatment because of a diagnosis of functional hearing loss. In total, 25 patients (13 women and 12 men) were treated in accordance with the study protocol, and data for assessment of the primary and secondary outcomes were available for all patients. The median age at registration was 49 years (range 23-72 years). Comorbidities were found in 22 of the 25 patients (88%), and 18 of the 25 patients (72%) had a history of previous diseases. None of the comorbidities or previous diseases presented were directly associated with SSHL. None of the patients had family histories of SSHL. All 25 patients complained of associated symptoms: 22 (88%) complained of tinnitus, 19 (76%) had a feeling of ear fullness and 14 (56%) complained of dizziness. The median interval between the onset of SSHL and the initiation of the test treatment was 23 days (range 15-32 days). The mean hearing level at registration was 81.2 dB (95% confidence interval (CI), 71.2 to 91.1).

A summary of the hearing recovery according to pure-tone audiometry for all of the patients is shown in Table 2. At 12 weeks after the test treatment, 48% (95% CI 28% to 69%; *P *= 0.086) of the patients showed hearing improvement. The null hypothesis for the primary outcome was not rejected. Of the 25 patients, 0 showed complete recovery, 1 (4%) showed marked recovery, 11 (44%) showed slight recovery and 13 (52%) showed no recovery at 12 weeks. None of the patients who were treated more than 26 days after the onset of SSHL showed hearing improvement. At 24 weeks after the test treatment, the proportion of patients showing hearing improvement was 56% (95% CI 35% to 76%; *P *= 0.015), showing that the null hypothesis was rejected for the data at 24 weeks. Of the 25 patients, none showed complete recovery, 1 (4%) showed marked recovery, 13 (52%) showed slight recovery, and 11 (44%) showed no recovery. Two patients showed a hearing improvement of less than 10 dB at 12 weeks after the treatment, but an improvement of 10 dB at 24 weeks.

**Table 2:** Hearing recovery according to pure-tone audiometry

				Averaged hearing level (dB)			Hearing improvement	
				
Patient	Age	Gender	Days from onset	Before registration	12 weeks	24 weeks	12 weeks	24 weeks
1	54	M	19	88	77	75	SR	SR

2	36	F	31	62	55	60	NR	NR

3	46	M	21	107	81	86	SR	SR

4	29	F	24	107	95	95	SR	SR

5	38	M	19	65	64	62	NR	NR

6	72	M	29	98	97	97	NR	NR

7	49	M	17	111	105	105	NR	NR

8	49	F	26	47	42	42	NR	NR

9	55	M	21	104	78	75	SR	SR

10	55	F	29	52	57	57	NR	NR

11	60	F	27	37	33	32	NR	NR

12	35	F	21	76	68	66	NR	SR

13	59	M	23	90	79	78	SR	SR

14	58	M	32	60	81	77	NR	NR

15	60	F	26	63	40	39	SR	SR

16	36	M	19	56	51	46	NR	SR

17	33	F	18	88	88	87	NR	NR

18	61	F	25	92	72	74	SR	SR

19	42	F	15	111	89	92	SR	SR

20	23	F	18	79	22	18	MR	MR

21	45	F	26	95	82	77	SR	SR

22	45	M	28	87	84	85	NR	NR

23	60	F	23	108	84	86	SR	SR

24	26	M	20	109	92	86	SR	SR

25	55	M	21	37	34	35	NR	NR

Average hearing level was the mean hearing level according to pure-tone audiometry at the five frequencies tested (0.25, 0.5, 1.0, 2.0 and 4.0 kHz). Hearing improvement was determined by the criteria shown in Table 1.

MR = marked recovery; NR = no recovery; SR = slight recovery.

No serious adverse events associated with the test treatment occurred, although any adverse events were recorded in all of 25 patients to be evaluated. Adverse events with an incidence rate of more than 20% included dizziness (44%), nausea (24%), otitis externa (32%), common cold (20%) and otitis media (28%). All adverse events disappeared within the observation period. Except for two patients, the dizziness appeared either on the day of local IGF1 application or on the next day, and continued for a mean of 6.4 days (range 1-20 days). In all patients, the dizziness appeared after the test treatment. In one patient, dizziness appeared 2 months after the test treatment and continued for 4 months. In another patient, dizziness appeared 7 days after the application and disappeared 2 days later. Otitis media was found in 7 of the 25 (28%) patients, and was cured within a mean of 9.4 days (range 2-17 days). Exacerbation of tinnitus appeared in two patients at 29 and 33 days after the test treatment, respectively. None of the patients showed residual perforation of the tympanic membrane or additional hearing loss over 10 dB.

## Discussion

Hearing loss is common, affecting about 5% to 6% of the population of the USA [1]. SSHL is one of the most common clinical conditions encountered by otolaryngologists, although it is less common than age-related hearing loss. National surveys have demonstrated the incidence of SSHL to be 5-30 per 100,000 per year [2,16,17]. Systemic application of glucocorticoids has been used as a standard therapy, although the supporting evidence is weak. Although systemic glucocorticoid application results in hearing recovery in some patients with SSHL, approximately 20% show no recovery [3]. Alternative therapeutic treatment options for SSHL have thus been eagerly sought. Against this background, we began developing topical IGF1 treatments using gelatin hydrogels in animal models [5,11,12], followed by a clinical trial to investigate their safety and efficacy for use in patients with SSHL. Some studies have indicated that SSHL develops when the inner ear does not receive a sufficient oxygen supply [18]. Consequently, hyperbaric oxygen treatment has been used as an alternative option for the treatment of SSHL [19,20]. At Kyoto University Hospital, hyperbaric oxygen therapy has been used as a secondary treatment of choice for glucocorticoid-resistant SSHL [14]. We thus used the proportion of patients with glucocorticoid-resistant SSHL showing hearing recovery following hyperbaric oxygen therapy as a historical control.

Here, we report hearing recovery according to pure-tone audiometry and incidence of adverse events following topical IGF1 application using gelatin hydrogels in patients with SSHL enrolled in a single arm, non-randomised and open trial. Topical IGF1 treatment resulted in hearing recovery in approximately half of the patients with SSHL that had not responded to systemic glucocorticoid application, although the null hypothesis was rejected at 24 weeks after the test treatment but not at 12 weeks. In addition, no serious adverse events were observed during the 24-week observation period. The results indicated that the topical IGF1 application using gelatin hydrogels was safe, and had equivalent or superior efficiency to the hyperbaric oxygen therapy that was used as a historical control; this suggests that the efficacy of topical IGF1 application using gelatin hydrogels for SSHL that is resistant to systemic glucocorticoid treatments should be evaluated using randomised clinical trials.

Spontaneous recovery occurs in 40% to 65% of patients with SSHL [21,22], which makes it difficult to examine the exact therapeutic effects of interventions. It is therefore important either to eliminate patients with spontaneous recovery from such trials or to include a placebo control. In the present study, the test treatment was initiated in all patients more than 14 days (mean 23 days; range 15-32 days) after the onset of SSHL. In most cases, spontaneous recovery occurs within 14 days of onset [21]. We therefore consider spontaneous recovery to have had a negligible influence on the present results.

As a secondary treatment of choice for SSHL, intratympanic injection of glucocorticoids has gained considerable attention, because it seems to deliver a high concentration of glucocorticoids to the inner ear [23]. In addition, local application can reduce the total amount of glucocorticoids that needs to be applied, leading to a reduced risk of adverse events [24]. However, this approach remains controversial, because the criteria used to judge its efficacy differ in the literature. Haynes *et al. *[25] reviewed the literature on the intratympanic injection of glucocorticoids for SSHL after the failure of systemic treatment, and re-estimated the hearing recovery based on their own criteria, according to which a 20 dB improvement as indicated by pure-tone audiometry or a 20% improvement in discrimination was considered to be a successful therapeutic intervention. The recovery rates according to their criteria were 0% to 40%. When these criteria for successful intervention were applied to the data from the present study, the recovery rate was 24%, suggesting that the efficacy of topical IGF1 treatment using gelatin hydrogels might be equivalent to that of the intratympanic injection of glucocorticoids. We therefore recommend that the efficacy of topical IGF1 treatment using gelatin hydrogels should be evaluated in a randomised clinical trial, and its effectiveness for SSHL should be compared with that of the intratympanic injection of glucocorticoids.

## Conclusions

The present results indicate the safety and efficacy of the use of topical IGF1 treatment using gelatin hydrogels for SSHL resistant to systemic glucocorticoid treatments. A double-blinded, randomised clinical study could clarify these findings. However, there are ethical obstacles to the use of double-blinded, randomised clinical trials for SSHL. For instance, the time from the onset of SSHL to the start of treatment has been regarded as important for the outcome, with prompt treatment preventing the development of irreversible auditory pathological changes. In addition, systemic glucocorticoid treatments have widely been accepted as a standard therapy for SSHL, and have led to improvement in some patients [26]. Hence, there would be ethical difficulties in not offering patients treatment with systemic glucocorticoids. Moreover, topical IGF1 application using gelatin hydrogels requires the use of surgical procedures, which would make it difficult to test in a double-blinded study. Therefore, as a next step, we will conduct a randomised clinical trial to compare the efficacy of topical IGF1 treatment using gelatin hydrogels with that of the intratympanic injection of glucocorticoids in patients with SSHL that is resistant to systemic glucocorticoids; it is hoped that this might clarify the efficacy of topical IGF1 treatment using gelatin hydrogels.

## Competing interests

The authors declare that they have no competing interests.

## Authors' contributions

TN, RO, SaT and JI planned the study. TS, HH, YSK and NM performed surgical treatment and collected the data. KH, KO, AY, KI, MY and YT prepared the gelatin hydrogels. SaT, ShT and HT analysed the data. TN wrote the article. JI edited the article.

## Pre-publication history

The pre-publication history for this paper can be accessed here:

http://www.biomedcentral.com/1741-7015/8/76/prepub
